# Effects of dexamethasone on intracochlear inflammation and residual hearing after cochleostomy: A comparison of administration routes

**DOI:** 10.1371/journal.pone.0195230

**Published:** 2018-03-30

**Authors:** Ah-Ra Lyu, Dong Hyun Kim, Seung Hun Lee, Dong-Sik Shin, Sun-Ae Shin, Yong-Ho Park

**Affiliations:** 1 Department of Otolaryngology-Head and Neck Surgery, College of Medicine, Chungnam National University, Daejeon, Republic of Korea; 2 Department of Medical Science, College of Medicine, Chungnam National University, Daejeon, Republic of Korea; 3 Biomedical Convergence Research Center, Chungnam National University Hospital, Daejeon, Republic of Korea; 4 Brain Research Institute, College of Medicine, Chungnam National University, Daejeon, Republic of Korea; University of Miami School of Medicine, UNITED STATES

## Abstract

Preservation of residual hearing after cochlear implant is an important issue with regards to hearing performance. Various methods of steroid administration have been widely used in clinical practice to reduce inflammation and preserve residual hearing. Here we compare the effect of different routes of dexamethasone administration on intracochlear inflammation and residual hearing in guinea pig ears. Dexamethasone was delivered into the guinea pigs either through intracochlear, intratympanic or systemic route. The intracochlear concentration of dexamethasone, residual hearing, inflammatory cytokines and histopathologic changes were evaluated over time. A higher intracochlear dexamethasone concentration was observed after intracochlear administration than through the other routes. Residual hearing was better preserved with local dexamethasone administration as was supported by the reduced inflammatory cytokines, more hair cell survival and less severe intracochlear fibrosis and ossification concurrently seen in the local delivery group than in the systemic group. The results demonstrate that local dexamethasone delivery can reduce intracochlear inflammation and preserve residual hearing better than in systemically administered dexamethasone.

## Introduction

Dexamethasone is a widely used steroid that has potent anti-inflammatory effect. Steroid is the treatment of choice for idiopathic sudden sensorineural hearing loss[1–3] and has been regarded as having a role in the preservation of residual hearing in cochlear implant surgery[4–6]. The expression of glucocorticoid receptor in the cochlea has become a convincing reason for steroid application in this area[7, 8]. Dexamethasone can be administered systemically or locally. Several reports have shown that dexamethasone, whether applied locally[9–13] or systemically[5, 14, 15], was able to significantly reduced hearing loss. It is still questionable whether systemic or intratympanic injected steroid can penetrate the cochlea effectively. From a more simplistic point of view of directly delivering a drug into the cochlea, an intracochlear injection seems to be the most straightforward. However, assessing drug level inside the cochlea requires a cochleostomy or puncture of the round window membrane, both surgical approaches would be, by themselves, traumatic to the cochlea. An atraumatic and effective method of steroid delivery into the cochlea are the main goals and it is still unknown which route of delivery would be most appropriate in achieving these. In this study, we aimed to compare the effects of dexamethasone administered via different routes on intracochlear inflammation and residual hearing after cochleostomy.

## Materials and methods

### 1. Animals

All animal experiments were approved by the Chungnam National University, Committee of the Animal Experiment (CNU00499). Ninety male albino guinea pigs, weighing 250-300g each, with normal hearing prior to surgery were enrolled in this study. Eighty-four animals were used for the experimental group with bilateral cochleostomy and this experimental group was divided into cochleostomy only group (CS), intracochlear dexamethasone group (IC), intratympanic dexamethasone group (IT) and intraperitoneal dexamethasone group (IP) (see below). Time point studies were performed (9 animals for 10, 30, and 90 minutes, 21 animals for 1 day, 12 animals for 3 days, 1 and 2 months). Thirty-six animals were used for perilymph sampling at each time point (10, 30, 90 minutes, and 1 day). Another 24 animals were used for real time polymerase chain reactions at each time point (1 and 3 days) and 24 animals were used for immunohistochemistry (30 days, n = 12) and sectional study (60 days, n = 12). The remaining 6 animals were used as normal controls for real time polymerase chain reactions (N = 3) and perilymph sampling (N = 3).

### 2. Surgical procedure for cochleostomy and dexamethasone application

The animals were anesthetized with intramuscular injection of combination of tiletamine HCl and zolazepam HCl 40 mg/kg (Zoletil, Virbac Animal Health, Carros, France) and xylazine 10 mg/kg (Rompun, Bayer Animal Health, Monheim, Germany). In addition, 0.5 ml of 1% lidocaine HCl was injected subcutaneously in the postauricular area for local anesthesia. The anesthetized animals were placed in a prone position on a thermoregulated heated pad. A retroauricular incision was made and blood was taken from an exposed neighboring blood vessel using an insulin syringe. The temporal bone was exposed and opened to visualize the round window membrane. A small cochleostomy was made in the bone near the round window with a sharp pick. The cochleostomy was made bilaterally. Using a micro cannula connected to the tip of a 30 gauge needle and Hamilton syringe (Hamilton Company, Reno, NV), 5μl of dexamethasone (5mg/ml, Huons, Korea) was injected into the scala tympani through the left cochleostomy site using an infusion pump for 2 minutes. The cochleostomy site and bulla were then sealed with tissue adhesive (Durelon, 3M ESPE, Germany) and carboxylate cement (Durelon, 3M ESPE, Germany) in IC group. Similar injection method described above was also employed in the IT group except for the tip of the cannula was instead directed into the tympanum to create a tympanic bullae filled with dexamethasone. Dexamethasone (10mg/kg) was injected intraperitoneally for 3 days in the IP group. The skin incision was closed in two layers. Afterwards, the animals were allowed to recover from anesthesia, and their pain was controlled with carprofen (Rimadyl, 4mg/kg, subcutaneously, Pfizer, NY, USA). No animal died in this set of experiments. Morbidity was limited to signs that are typical after cochleostomy, including unsteadiness and occasional head tilt. These resolved within a few days and did not worsen.

### 3. Auditory brainstem response

The hearing threshold shifts after surgery were evaluated in each group via auditory brainstem response (ABR) threshold at 4, 8, 16, 23 kHz, and click sound. The ABRs were recorded prior to surgery, just after surgery, at 7days, 1 and 2 months after surgery. TDT System-3 (Tucker Davies Technologies, Gainseville, FL, USA) hardware and software were used to obtain the ABRs. The stimuli were computer generated tone pips. Subcutaneous needle electrodes were placed around the skull vertex and both infra-auricular areas. Tone bursts with duration of 4 ms and a rise-fall time of 1 ms at frequencies of 4, 8, 16, 32 kHz, and clicks were used. The sound intensity was varied by 10-dB intervals for the tone-burst sounds and by 5 dB intervals for the click sounds near the threshold. The waveforms were analyzed using a custom program (BioSig RP, ver. 4.4.1) with the researcher blinded to the treatment group. Threshold was defined as the lowest stimulus intensity to evoke a wave III response greater than 0.2 mV.

Further ABR threshold measurements were done at 7 days, 1 and 2 months after the operation. The differences in ABR thresholds were averaged across the frequency range for each cochlea to yield their individual mean rise in ABR threshold. Threshold shift was defined as the difference between preoperative and one of the postoperative values. A positive threshold shift indicated an elevation of the auditory threshold.

### 4. Cochlear fluid sampling and analysis

The change of dexamethasone concentration over time in the cochlea in each group was determined by measuring the amount of drug in the perilymph fluid collected from the cochlear apex done at 10, 30, 90 minutes and 1 day after surgery, as detailed previously[16]. In brief, the tympanic bulla was washed several times with lactated Ringer’s solution and suctioned out. After removal of cochlear apex mucosa, small apical cochleostomy was done with a sharp pick and about 3~4μl perilymph was collected in hand-held graduated glass capillary tubes (IntraMARK micropipettes, BRAUBAND, Wertheim, Germany) marked at every 0.5μl volume. Each cochlear fluid sample was analyzed using mass system (QTRAP 4000, Applied Biosystems, Carlsbad, CA) interfaced with high-performance liquid chromatography (LC-MS/MS, Agilent 1260, Agilent Technologies, Santa Clara, CA) to measure the dexamethasone concentration in the perilymph. Samples were injected into a C18 column (XBD C18, 50 mm × 2.1 mm; Agilent Technologies, Santa Clara, CA) with a mobile phase of acetonitrile:water (50:50, v/v) ran at a flow rate of 0.4 ml/min.

### 5. Quantitative real time polymerase chain reaction

Comparison of the early inflammatory responses between groups was done by sacrificing the animals at either 1 day or 3 days after surgery and quantitative real time polymerase chain reactions (qRT-PCR) for IL-1β, IL-6, TNF-α, and NOS2 were conducted. Interleukin-1β (IL-1β), interleukin-6 (IL-6), tumor necrosis factor-α (TNF-α) and nitric oxide synthase 2 (NOS 2) were measured and used as indicators of inflammatory response. Dissected cochleae were ground in 1 ml of TRIZOL reagent (Invitrogen, Carlsbad, CA, USA), 200 μl of chloroform was added, mixed gently and then centrifuged at 13,000 rpm for 15 minutes. About 450 μl of supernatant was transferred to a fresh tube and the same amount of isopropanol was added, shaken for 5 minutes, and centrifuged at 13,000 rpm for 15 minutes. The resulting pellet was suspended in 1 ml of 80% ethanol (in DEPC-treated water) and centrifuged at 13,000 rpm for 15 minutes. The same procedure was performed one more time and the pellet was then washed with 100% ethanol repeatedly. RNA was dissolved in 20 μl of RNase-free water. The purified RNA was quantified using Nano drop (NanoDrop Technologies Inc., Wilmington, DE, USA) by measuring UV absorbance of 260 nm. A total of 13 μl of RNA (2 μg each) with oligo-dT primer and DEPC-treated water was pre-denatured for 10 minutes at 65°C followed by addition of 4 μl of 5x reaction buffer, 2 μl of dNTP, 0.5 μl of RNase inhibitor, and 0.5 μl of RTase. The mixture was reverse transcribed for 1 hour at 50°C and 5 minutes at 85°C with the cDNA Synthesis Kit (Roche, IN, USA). The real-time reverse transcription process was performed according to the manufacturer’s procedure with SYBRgreen (Invitrogen, Grand Island, NY, USA). Comparative quantification of IL-1β, IL-6, TNF-α, and NOS 2 mRNA was obtained by comparative cycle of the threshold method. The quantitative RT-PCR was performed 3 times for each sample. The details of primers used in the polymerase chain reaction to detect IL-1β, IL-6, TNF-α, and NOS 2 are presented in Table 1.

**Table 1:** Primers used in quantitative real time polymerase chain reaction to detect IL-1β, IL-6, TNF-α, and NOS2.

Primer name		Sequence (5'-3')
GAPDH	Forward	5'-GCCCTCAATGACCACTTTGT-3'
	Reverse	5'-TGCTGTAGCCGAACTCATTG-3'
IL-1β	Forward	5'-TCCCTGTGAAAACAAGAGCA-3'
	Reverse	5'-CGCCTTTCTCTTGGAGCTTA-3'
IL-6	Forward	5'-AATTCCTGAGCCCAACTCCA-3'
	Reverse	5'-TGCTTTCCGAATAGCCCTCA-3'
TNF-α	Forward	5'-ATCAAGAGTCCCTGCCAGAA-3'
	Reverse	5'-CTCCCAGGTAGATGGGTTCA-3'
NOS2	Forward	5'-CCCTCTTCGTGCTGAAAAAG-3'
	Reverse	5'-GTCATGAGCAAAGGCACAGA-3'

### 6. Tissue preparation and immunohistochemistry

Selected animals were sacrificed at 1 month after surgery and cochlear tissues were obtained to assess survival of hair cells and nerve fibers. Tissues were fixed in 4% paraformaldehyde in PBS for 1 hour at room temperature. The cochlear bony walls and lateral wall tissues were first removed and the remaining cochlear tissues were prepared for immunostaining. Tissues were permeated with 0.3% Triton X-100 (Sigma–Aldrich Co., St. Louis, MO) for 10 minutes, blocked in 5% normal goat serum (Vector Laboratories, Inc., Burlingame, CA) for 30 minutes and were then incubated with rabbit anti-myosin VIIa primary antibody (Proteus BioSciences, Inc., Ramona, CA) and mouse anti-NF200 primary antibody (Novus Biologicals, Littleton, CO) at a concentration of 1:200 in blocking solution overnight at 4°C. After rinsing in PBS for 10 minutes, the tissues were incubated with the corresponding AlexaFluor 594 goat anti-rabbit secondary antibody (Molecular Probes, Eugene, OR) or AlexaFluor 488 goat anti-mouse secondary antibody (Molecular Probes, Eugene, OR) at a concentration of 1:200 in PBS for 30 minutes. After rinsing in PBS for 10 minutes, specimens were further dissected to separate individual cochlear turns and mounted on glass slides using CrystalMount (Biomeda, Foster City, CA). The specimens were observed using an epifluorescence microscope (Zeiss Axio Scope A1; Zeiss, Germany) with digital camera and the surviving hair cells were counted in each 100 μm of tissue.

**Fig 1:**
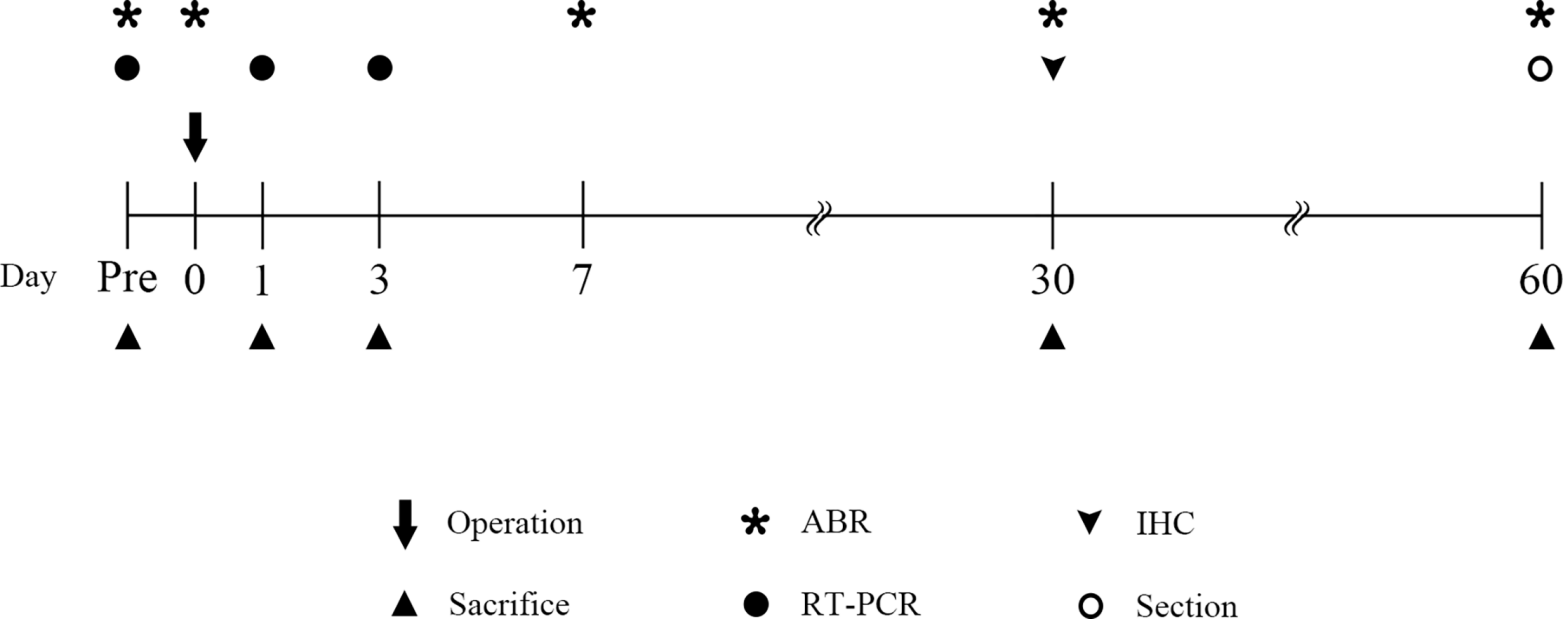
Schematic time-line of experiments. ABR thresholds were measured at prior to surgery, just after surgery, 2 days, 1 and 2 months after surgery. qRT-PCR were conducted at 1 and 3 days after surgery and control animal for the evaluation of acute inflammatory responses. Assessment of hair cells and nerve fiber survival were evaluated at 1 month after surgery. Intracochlear histopathologic changes were evaluated at 2 months after surgery.

Both cochleae were harvested from the animals 2 months after the operation for sectional study to assess intracochlear fibrosis and ossification. The harvested samples were placed in 4% paraformaldehyde in PBS for 2 hours, decalcified in EDTA (ethylene diamine tetra-acetic acid, 5% nitric acid) for 3 weeks, embedded in paraffin, sectioned in the mid-modiolar plane at a thickness of 5 μm and stained with hematoxylin and eosin. The stained tissue sections were examined and representative fields photographed using a light microscope (Olympus BX51; Olympus, Tokyo, Japan). All histologic sections were examined for evidence of intracochlear fibrosis and new bone formation. The timeline for all experiments are shown in Fig 1.

### 7. Image processing and statistical analysis

Adjustment of image contrast, superimposition of images, and colorization of monochrome fluorescence images were performed using Adobe Photoshop (version 7.0). Statistical analysis was performed with Graphpad Prism (version 3.02, San Diego, CA, USA) and SPSS (version 16.0, SPSS Inc., Chicago, IL). ABR threshold shift and the levels of inflammatory cytokine data taken before and after the surgery in each group were compared using One-way repeated measure ANOVA and the differences between groups at each time point were compared using one-way ANOVA. The hair cell survival between groups were compared using Kruskall-Wallis test. *p* values of < 0.05 were considered significant.

## Results

### 1. ABR threshold shifts

**Fig 2:**
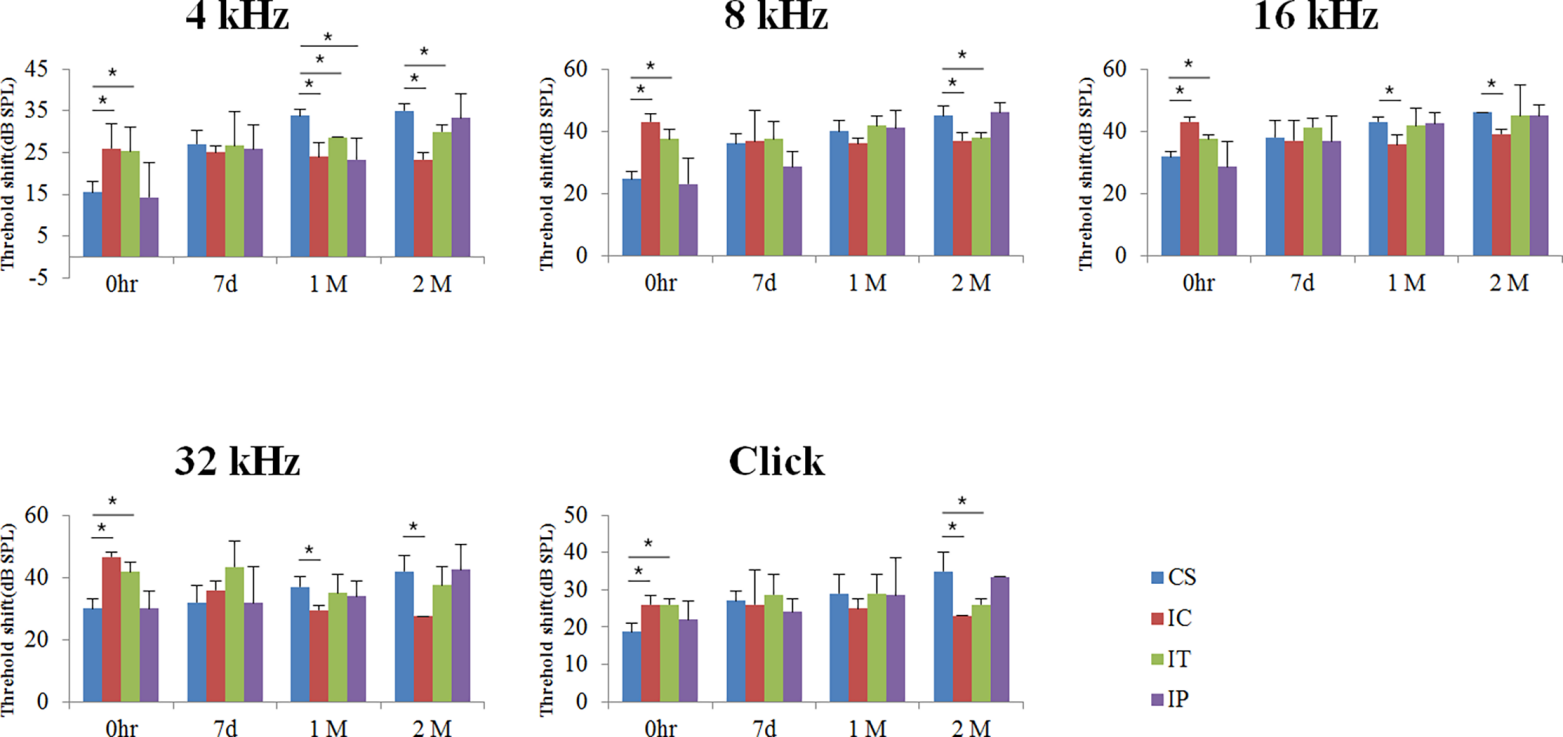
Auditory brainstem response threshold shifts at just after surgery, 7 days, 1 and 2 months after surgery. ABR threshold shifts were greater in the IC and IT groups compared to the CS and IP groups at just after surgery and lesser in the IC group compared to the other groups at 2 months after surgery. Asterisk indicates p<0.05.

ABR threshold shifts were increased in the CS group with the passage of time and these threshold shifts were greater in the IC and IT groups at just after surgery in all measured frequencies compared to the CS and IP groups at just after surgery. In the IC group, the differences were significant with 4, 16, 32 kHz and click at 1 month after surgery and all measured frequencies at 2 months after surgery compared to CS group (p<0.05). In the IT group, the differences were significant with 4 kHz at 1 month after surgery and 4, 8 kHz and click at 2 months after surgery compared to CS group (p<0.05). The threshold shifts in the IP group showed similar pattern with no significant differences compared to the CS group. These suggested that residual hearing was better preserved in the IC and IT groups compared to the CS and IP groups (Fig 2).

### 2. Changes of intracochlear dexamethasone concentration

**Fig 3:**
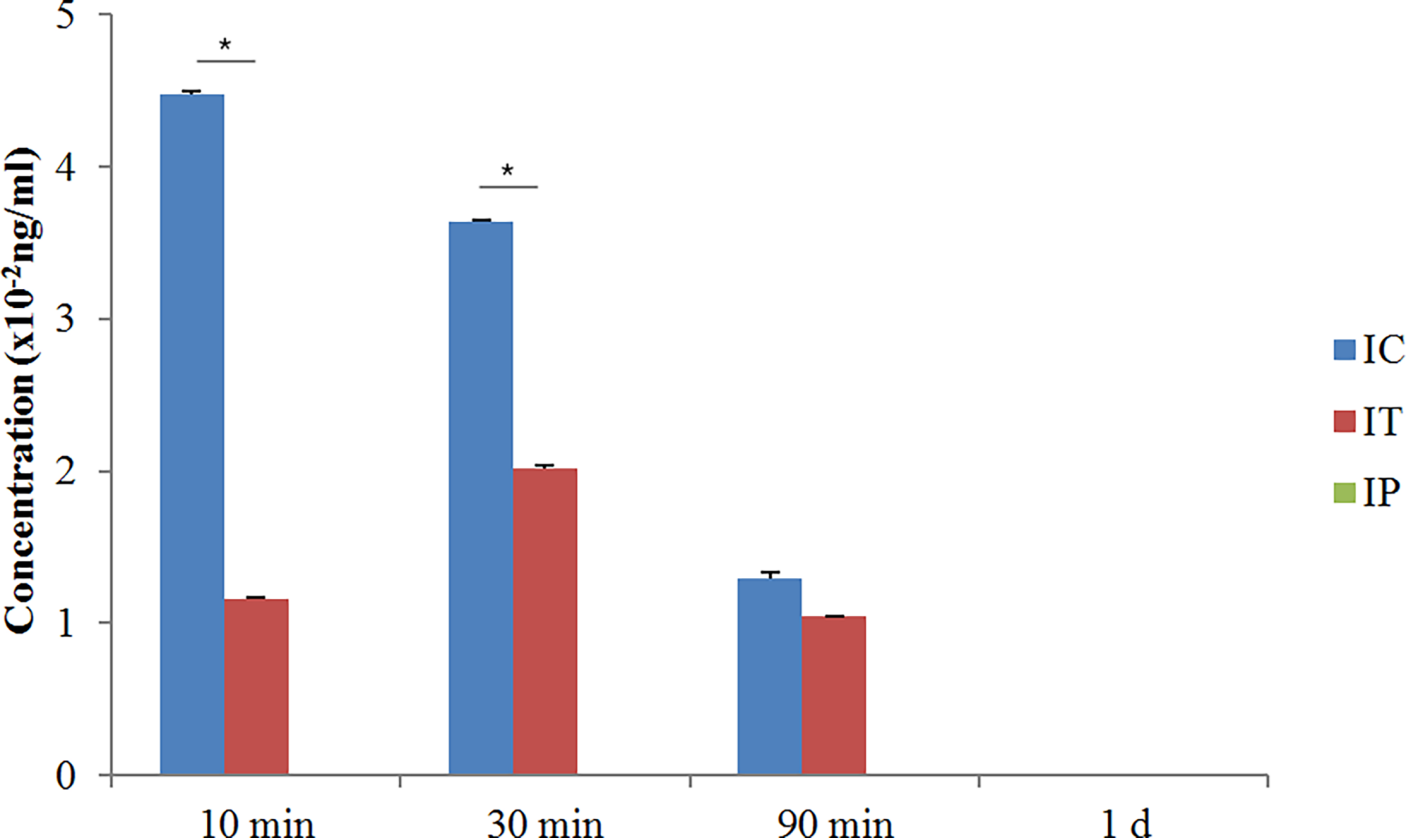
Concentration of dexamethasone in the perilymph after surgery over time. Concentration of dexamethasone was highest at 10 minutes in the IC group and at 30 minutes in the IT group. It was significantly higher in the IC group compared to the other groups at 10 and 30 minutes after surgery. Dexamethasone was not detected at all measured time points in the IP group and 1 day after in all group. Asterisk indicates p<0.05.

The concentration of dexamethasone in the cochlea was highest at 10 minutes in the IC group and at 30 minutes in the IT group. It showed a declining pattern over time. Dexamethasone was not detectable in the cochlea in the IP group at any measured time points. The concentration of dexamethasone was significantly higher in the IC group at 10 and 30 minutes compared to the IT group (p<0.05). This suggested that the IC route was a more effective method for dexamethasone delivery into the cochlea than via the IT and IP routes (Fig 3).

### 3. Changes of inflammatory cytokine

**Fig 4:**
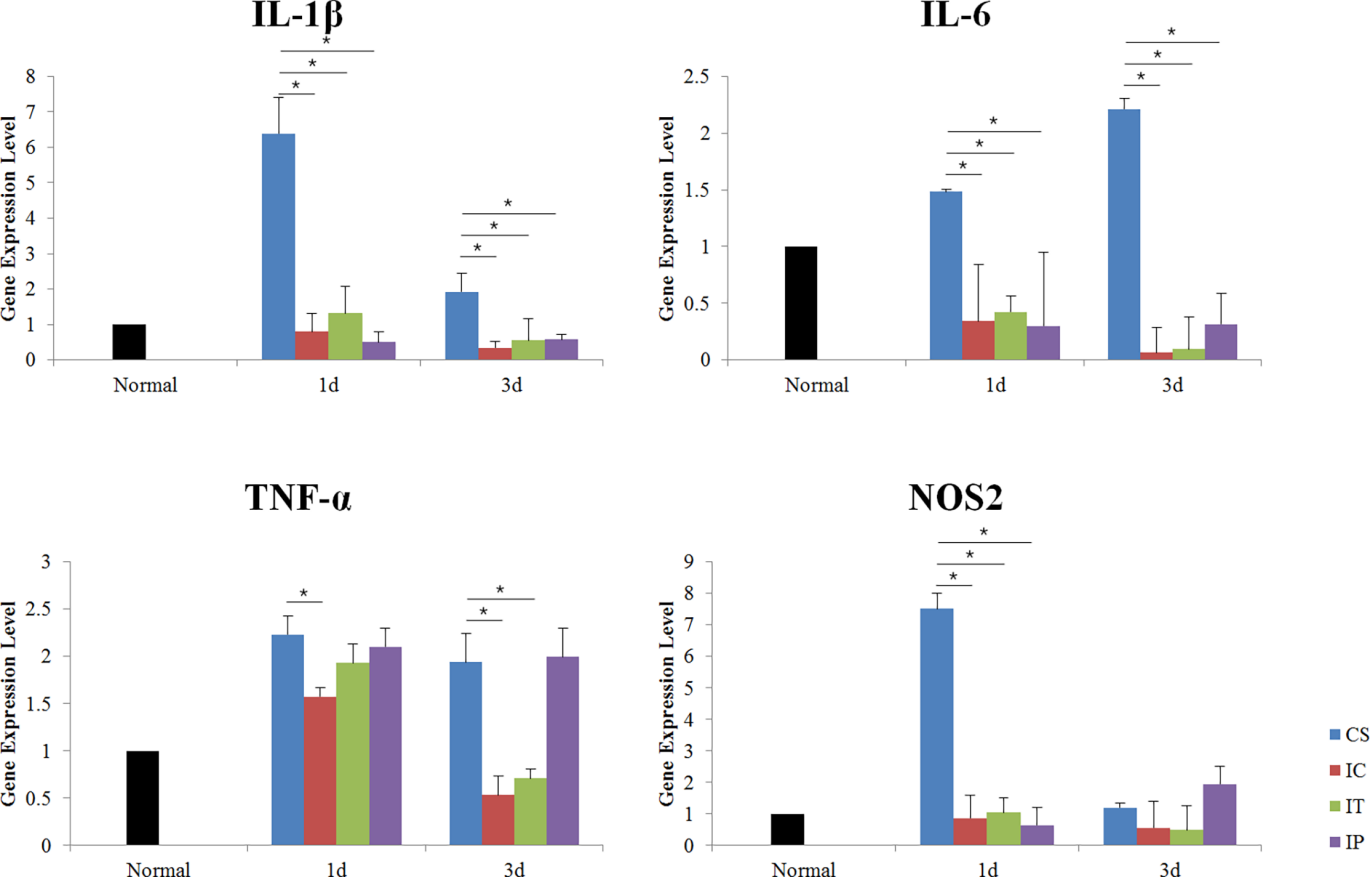
Quantitative real time polymerase chain reactions at 1 and 3 days after surgery. IL-1β, IL-6, and NOS2 were significantly increased in CS group compared to the other groups at 1 day after surgery and the increased IL-1β and IL-6 were sustained until 3 days after surgery. TNF-α was significantly decreased in the IC group at 1 day and in the IC and IT groups after surgery compared to other group. The increased TNF-α was sustained until 3 days after surgery in the IP group. Asterisk indicates p<0.05.

IL-1β, IL-6, TNF-α and NOS2 were significantly increased in the CS group compared to the normal ear at 1 day and the increase of IL-1β, IL-6, TNF-α were sustained until 3 days after surgery. IL-1β, IL-6 and NOS2 were significantly decreased in the IC, IT and IP groups compared to the CS group, while TNF-α was decreased in the IC and IP groups compared to the CS group at 1 day after surgery (p<0.05). At 3 days after surgery, IL-1β, IL-6 and NOS2 were significantly decreased in the IC, IT and IP groups compared to the CS group, while TNF-α was decreased in the IC and IT groups compared to the CS and IP groups (p<0.05). These suggested that the inflammatory responses in the cochlea were decreased, albeit with different patterns during the early stage, in the dexamethasone treated groups compared to the CS group (Fig 4).

### 4. Survival of hair cells

**Fig 5:**
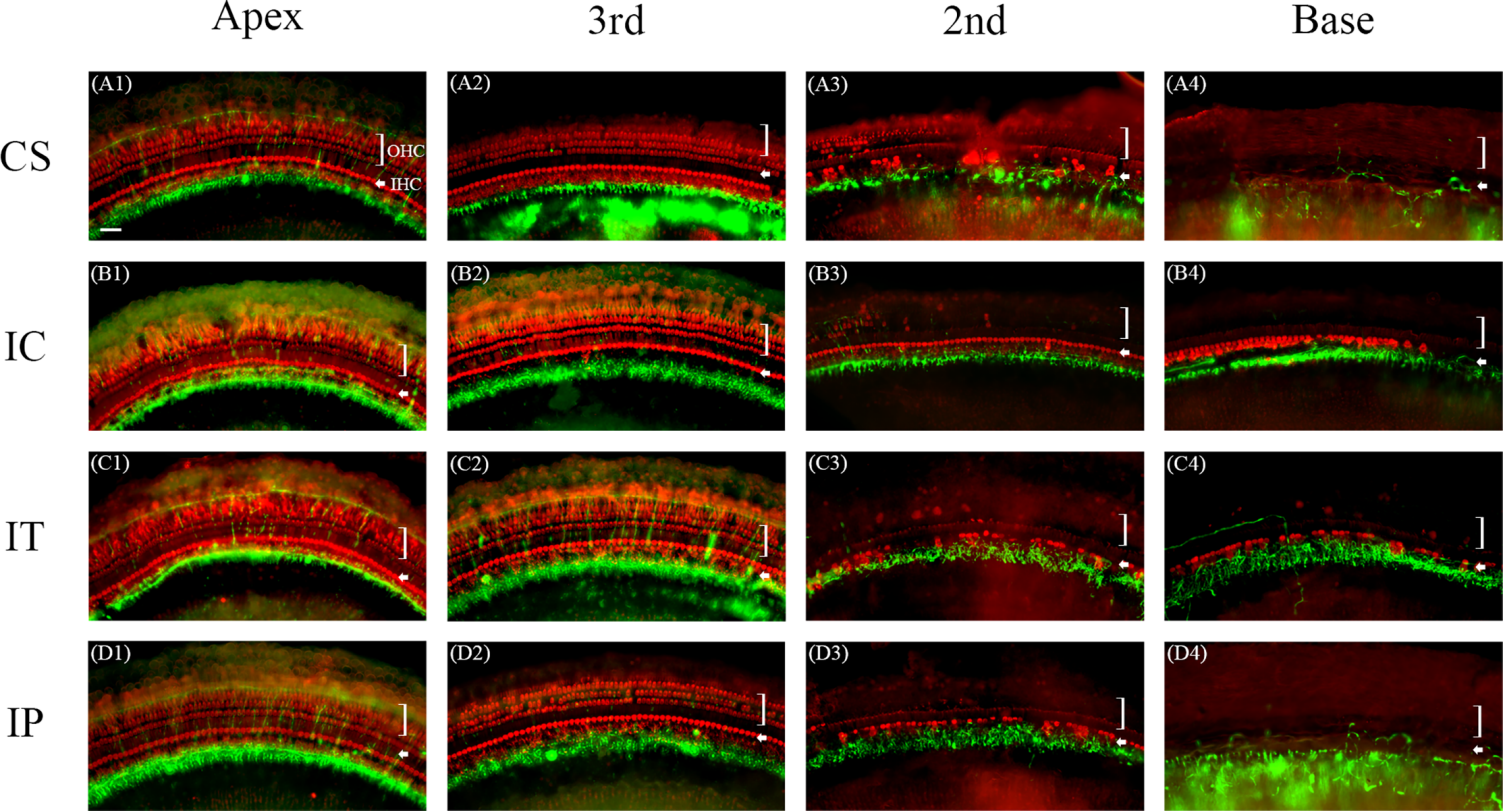
Whole-mounts of the auditory epithelium in CS (A1, A2, A3 and A4), IC (B1, B2, B3 and B4), IT (C1, C2, C3 and C4) and IP (D1, D2, D3 and D4) group at 1 month after surgery. Tissues were stained for myosin VIIa (red) to highlight the hair cells and NF-200 (green) for nerve fibers and then photographed with epifluorescence. Hair cell loss was more severe in the basal turn of the CS (A4) and IP groups (D4) compared to the IC (B4) and IT groups (C4). A1, B1, C1 and D1: Apical turn, A2, B2, C2 and D2: 3rd turn, A3, B3, C3 and D3: 2nd turn, A4, B4, C4 and D4: basal turn, OHC: outer hair cell, IHC: inner hair cell, Scale bar = 30 μm.

**Fig 6:**
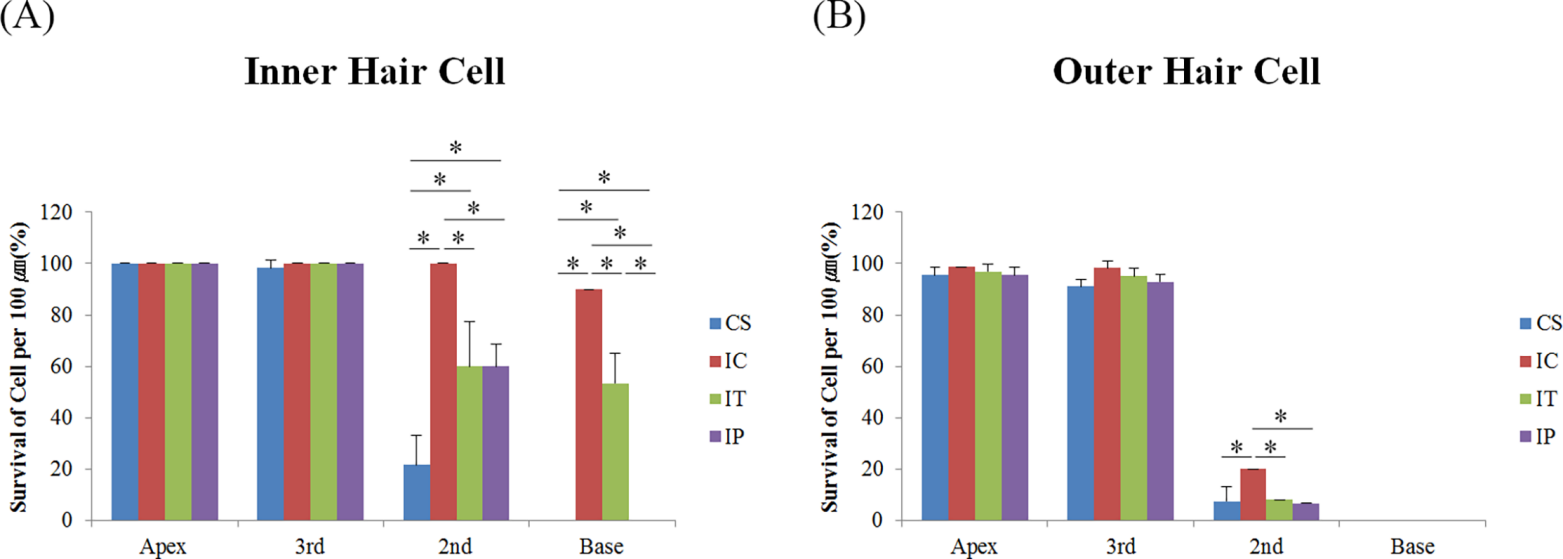
Survived hair cell counts after surgery. IHC were better preserved in the 2nd turn of the IC, IT and IP groups compared to the CS group and in the basal turn of the IC and IT groups compared to the CS and IP groups. IHC survival was significantly higher in the 2nd and basal turn of the IC group compared to other groups (A) and OHCs were better preserved in the 2nd turn of the IC group compared to other groups (B). Asterisk indicates p<0.05.

At 1 month after surgery, almost all the outer hair cells (OHC) were destroyed in the basal turn of the cochlea in all groups but more inner hair cells (IHC) survived in the basal turn in the IC and IT groups compared to the CS and IP groups (Fig 5A4, 5B4, 5C4 and 5D4). Hair cell counts also showed more surviving IHCs in the 2nd turn of the cochlea in the IC, IT and IP groups and in the basal turn in the IC and IT groups. This was significantly higher in the IC group compared to the other groups (Fig 6A). OHC were better preserved in the 2nd turn of the cochlea in the IC group compared to the other groups (Fig 6B). These suggested that the auditory HCs were better preserved in the IC and IT groups compared to the CS and IP groups. It appears that dexamethasone delivered via the IC route was superior over the other methods of delivery with regards to HC survival.

### 5. Intracochlear histopathologic changes

**Fig 7:**
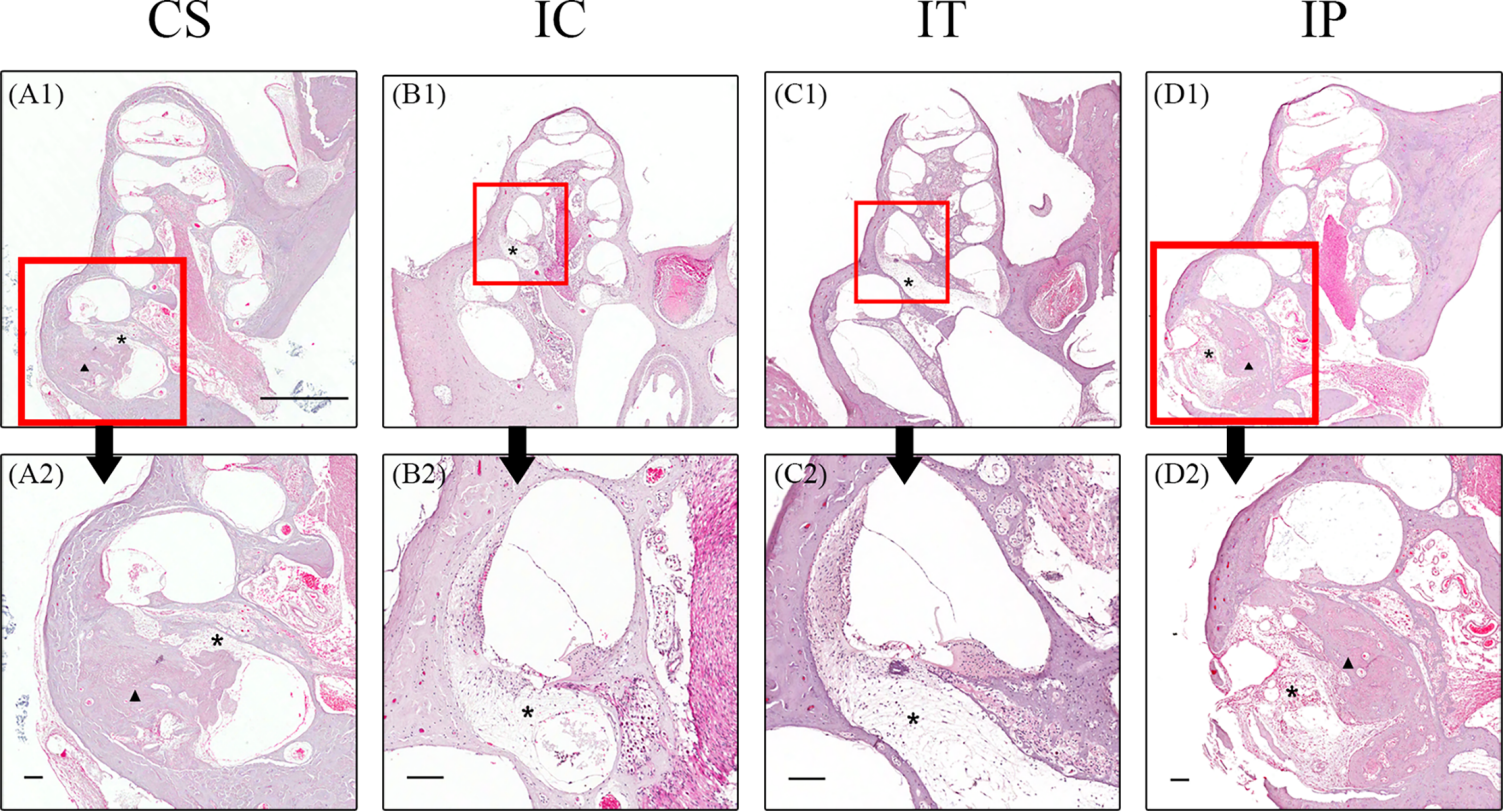
Histopathology of the cochlea in the CS (A1 and A2), IC (B1and B2), IT (C1 and C2) and IP groups (D1 and D2) at 2 months after surgery. More extensive ossification (arrow head) and fibrosis (asterisk) were observed in the basal turn of the CS (A1 and A2) and IP groups (D1 and D2) than the IC (B1and B2) and IT (C1 and C2) groups. Scale bar = 1 mm in A1, and 100 μm in A2, B2, C2 and D2.

Extensive ossification and fibrosis were observed in the basal turn of the cochlea in the CS (Fig 7A1 and 7A2) and IP groups (Fig 7D1 and 7D2) at 2 months after surgery. In contrast, only some fibrosis in the scala tympani without extensive ossification in the cochlea was observed in the IC (Fig 7B1 and 7B2) and IT groups (Fig 7C1 and 7C2). This suggested that inflammatory tissue response in the cochlea was less severe in the IC and IT groups compared to the CS and IP groups.

## Discussion

Although cochlear implant (CI) can restore hearing even in deaf patients, preservation of residual hearing in CI is still a quite important issue. There were several reports that the delayed reduction of CI benefits with the passage of time[17, 18], due to direct injury to the cochlea during surgery or delayed intracochlear fibrosis and ossification, may lead to residual hearing loss[19]. Furthermore, when there is a need for electro-acoustic stimulation, a method using acoustic stimulation of naive hearing with hearing aid in low frequency and electric stimulation in high frequency, the preservation of residual hearing is mandatory. An atraumatic surgery aimed at residual hearing preservation would be a very important issue for CI.

With the purpose of minimizing trauma to the cochlea in mind, surgeries have changed from the traditional cochleostomy to the less traumatic round window approach[20–23] and softer atraumatic electrodes have been developed[24, 25]. Furthermore, perioperative use of steroid has gained interest. Trials to deliver the steroid with dexamethasone eluting electrode have been considered[26–30] and other potentially more effective drug delivery methods into the cochlea, such as gel and nanoparticles, are being developed [31–34]. There have been several reports about hearing preservation with steroid treatment in animal studies. Although the administration methods and dosage of dexamethasone were somewhat different, many of them showed hearing preservation and reduced adverse tissue responses such as fibrosis and ossification with dexamethasone use[10–12, 27, 31, 32, 35–38]. The same outcome was also observed in our experiment. Hearing preservation was observed in all measured frequencies at 2 months after the surgery especially in the IC group compared to the CS, IT and IP groups. The immediate hearing losses observe were thought to be due to the loss of inner ear homeostasis such as inner ear hydrops in the IC group or the bullous space occupied by the dexamethasone in the IT group. Histopathology showed that more hair cells survived in the ear of the IC, IT and IP groups compared to the CS group. A recent human study by Cho HS et al demonstrated that the use of preoperative systemic and intraoperative topical steroid can offer better hearing preservation compared to not using steroid and that the use of perioperative steroid can help minimize inner ear damage after CI[5]. Rajan GP et al also showed that preoperative intratympanic methyl prednisone (glucocorticoid) can improve and stabilize hearing preserved after CI[4]. It seems that the use of steroid in CI surgery is being widely considered.

Steroid can be administered either systemically or locally. Local routes of steroid administration targeting the cochlea can be done via intratympanic or intracochlear methods. Theoretically, a direct intracochlear steroid delivery would result in the highest concentration of the medicine in the inner ear than the other routes. A local route also significantly minimizes the possibility of systemic side effects that often occur in systemically administrated drugs. Unfortunately, this direct intracochlear delivery is currently possible only through a cochleostomy or by puncturing the round window membrane during CI surgery; both surgical approaches are traumatic to the cochlea which by themselves may cause residual hearing loss. According to Bird PA et al, administration of dexamethasone via the intratympanic route resulted in a much higher perilymph drug concentration and much lower plasma concentrations compared with systemic administration in their human study[14]. It is still doubtful whether steroid can effectively cross the round window membrane or oval window in the intratympanic route or the blood labyrinth barrier in the systemic route to reach the cochlea and achieve therapeutic levels [39, 40]. In this study, we compared the intracochlear dexamethasone concentration in relation to the different administration routes against the passage of time. The highest concentration was observed in the IC group compared to the IT and IP groups, and it was not even detectable in the IP group until 1 day after surgery. This means that from the point of view of steroid concentration in the inner ear, the IC route appears to be more effective than the other routes. Moreover, there is no guarantee that systemically administrated dexamethasone can go through the blood labyrinth barrier and reach the inner ear effectively. Dexamethasone was not detected in the cochlea until 1 day after surgery in the IP group even though we administrated about 10 times the concentration (10mg/kg) of what is normally used clinically (1mg/kg) for 3 days.

Cochleostomy, in itself, can cause injury or trauma and is known to induce local inflammatory cytokine production which can lead to apoptosis of hair cells through the oxidative stress pathway and trigger immune cell recruitment into the cochlea[41–47]. It is believed that a high concentration of steroid in the cochlea may be feasible in local intracochlear inflammation control. This idea was supported when it was observed that immune response and inflammatory associated genes in the cochlea were down regulated with the use of dexamethasone-eluting electrode and a high intracochlear concentration of dexamethasone was able to reduce fibrosis around the electrode and impedance[38]. However, there was a report showing that despite of the better hearing preservation obtained with local steroid administration, this did not significantly reduce the inflammatory tissue volumes compared to systemic steroid. It was postulated that the steroid induced reduction in intracochlear injury signaling was insufficient to prevent immune cell recruitment into the cochlea[15]. In our study, although we did not test for immune cell recruitment or severity of systemic inflammation, all tested inflammatory cytokines in the cochlea were significantly reduced in the IC and IT groups. With the reduction of TNF-α in the IP group not occurring until 3 days after the surgery, it can be inferred that IP dexamethasone may be less effective compared to the IC and IT routes. This coincided with other reports showing that TNF-α induced hair cell loss[41, 48]. We believe that reducing both intracochlear and systemic inflammation may be important and that IP dexamethasone administration was not effective in reducing intracochlear inflammation.

This study did not investigate and compare the direct effect or injury of an inserted electrode upon the cochlear with the effect of different routes of dexamethasone administration on intracochlear inflammation and residual hearing after cochleosotmy. A higher dexamethasone concentration in the inner ear was observed with the direct intracochlear route than the other routes of administration and the intracochlear inflammation was lesser in the local routes than the systemic route. Residual hearing was better preserved with direct intracochlear dexamethasone administration and histopathologic studies supported this result. Further studies such as combined local and systemic administration and simultaneous evaluation of systemic inflammatory response is needed for a more detailed understanding of the mechanisms involved in controlling intracochlear inflammation.

## Conclusion

The direct intracochlear delivery route resulted in a higher dexamethasone concentration in the inner ear and lesser intracochlear inflammatory response leading to better hearing preservation than with intratympanic and systemic administration.
